# Mobile Manipulation Integrating Enhanced AMCL High-Precision Location and Dynamic Tracking Grasp

**DOI:** 10.3390/s20226697

**Published:** 2020-11-23

**Authors:** Huaidong Zhou, Wusheng Chou, Wanchen Tuo, Yongfeng Rong, Song Xu

**Affiliations:** 1 Robotics Institute, School of Mechanical Engineering and Automation, Beihang University, Beijing 100191, China; hdzhou@buaa.edu.cn (H.Z.); tuowanchen@buaa.edu.cn (W.T.); rongyongfeng2014@126.com (Y.R.); keithxs@buaa.edu.cn (S.X.); 2 State Key Laboratory of Virtual Reality Technology and Systems, Beihang University, Beijing 100191, China

**Keywords:** mobile manipulation, object detection, visual servo, dynamic tracking grasp, triangle match

## Abstract

Mobile manipulation, which has more flexibility than fixed-base manipulation, has always been an important topic in the field of robotics. However, for sophisticated operation in complex environments, efficient localization and dynamic tracking grasp still face enormous challenges. To address these challenges, this paper proposes a mobile manipulation method integrating laser-reflector-enhanced adaptive Monte Carlo localization (AMCL) algorithm and a dynamic tracking and grasping algorithm. First, by fusing the information of laser-reflector landmarks to adjust the weight of particles in AMCL, the localization accuracy of mobile platforms can be improved. Second, deep-learning-based multiple-object detection and visual servo are exploited to efficiently track and grasp dynamic objects. Then, a mobile manipulation system integrating the above two algorithms into a robotic with a 6-degrees-of-freedom (DOF) operation arm is implemented in an indoor environment. Technical components, including localization, multiple-object detection, dynamic tracking grasp, and the integrated system, are all verified in real-world scenarios. Experimental results demonstrate the efficacy and superiority of our method.

## 1. Introduction

Mobile manipulation is an important issue in the field of robotics. Traditionally, though the breaking down of sophisticated tasks into many single tasks, fixed-base manipulators, such as palletizing robots [1] and assembly robots [2], can complete tasks with high precision via pre-programming. Emerging applications in the industry, such as material handling and mobile welding, demand high mobility and flexibility of robots. A considerable amount of literature has grown up around the theme of mobile manipulation in recent decades [3].

With the developments of robotic technology and artificial intelligence, mobile manipulation has become increasingly practical. In a typical mobile manipulating process, the mobile manipulator should autonomously and safely reach the operating space, avoiding obstacles and achieving self-localization during movement. Then, it will proactively detect and locate the target and complete the grasping task. On the one hand, external sensors (e.g., laser radars) and simultaneous localization and mapping (SLAM) algorithms facilitate the autonomous navigation and localization of mobile robots [4]. The adaptive Monte Carlo localization (AMCL) algorithm has been proved to be an efficient probabilistic localization method [5]. On the other hand, with the assistance of deep learning, more environmental information can be extracted from the vision sensors, and the robot’s ability to perceive the environment is enhanced [6]; thus, the accuracy of object detection has been greatly improved. Accordingly, mobile manipulation can provide highly flexible services and dexterous operations in structured and unstructured environments. Autonomous mobile manipulators are gradually used in many fields, such as various industries [7], living spaces [8,9,10], and agriculture [11,12].

However, some complex tasks pose huge challenges for mobile manipulation. First, high-precision localization is critical for sophisticated work such as fixed-point operation and material handling in a warehouse environment. Existing approaches need to further improve the localization accuracy and cannot handle the kidnapping problem well, especially in highly similar environments. Furthermore, in dynamic scenarios (e.g., an assembly line), the objects to be grasped are usually diverse and moving. How to rapidly locate and accurately grasp these dynamic objects based on visual information is another important issue, which remains to be further studied [13].

To address the above problems, in this paper, we present a mobile manipulation system capable of performing high-precision localization and dynamic tracking grasp. To accomplish this task, we decompose the system into two subsystems: the navigation subsystem and the grasp subsystem. By using triangle-matched laser-reflector landmarks to continuously improve the weight and accuracy of the particles in AMCL, the navigation subsystem realizes rapid navigation toward the target area and achieves high-precision positioning. The grasp subsystem dynamically grasps moving objects by visual servo, which uses a deep-learning model for real-time object detection and uses the closed-loop feedback of the image position to achieve dynamic object tracking. The main contributions of this work are provided as follows:

(1) The localization accuracy of the AMCL algorithm is improved by adaptively adjusting the weight of particles based on the laser-reflector information.
(2) Efficient dynamic grasping is realized by exploiting real-time object-detection results to achieve closed-loop tracking.
(3) An integrated system containing hardware and software is developed and tested in real-world environments, which is capable of autonomous high-precision localization and dynamic object grasping. The key algorithm components and the complete system are both verified.

The paper is organized as follows. In Section 2, related work on mobile manipulation is reviewed. Section 3 presents the improved localization algorithm and the dynamic grasping policy. In Section 4, real-world experiments of mobile manipulation are described and discussed. Section 5 draws the conclusions and future work.

## 2. Related Work

Mobile manipulation is a fundamental problem in the field of robotics, and researchers are conducting extensive research on this problem. In the following section, we discuss and review some of the work related to mobile manipulation and our methods.

### 2.1. Mobile Manipulation

Extensive research on mobile manipulation has been conducted in many robot subdivisions, such as humanoid robots [14,15], wheeled robots [7,16], legged robots [17,18], unmanned aerial vehicles [19,20,21] and underwater robots [22]. In recent years, a large number of competitions related to mobile operations have been held, such as the Amazon Robotics Challenge [23,24], the DARPA Robotics Challenge [25], and the FetchIt! Mobile Manipulation Challenge [26]. The results of these challenges and projects suggest that the research and application of mobile manipulation is a logical integration of operation, navigation, perception, self-learning and multi-robot collaboration; therefore, we would expect that a mobile manipulators should be able to perform complex tasks in both structured and unstructured environments.

Many mobile manipulators are designed to handle complex tasks in different scenarios. Human Support Robot (HSR) [27] is a real home robot that can be used in a real home environment. The robot can accomplish the Tidy-Up task independently according to the layout of the furniture and the environment information given in advance. In [28], in order to overcome the system uncertainties and anomalies inherent in autonomous mobile manipulator, a coordinated control strategy combining visual and force servos into a sophisticated reactive task control is proposed, and this control strategy achieved high reliability in the “peg-in-hole” type of insertion assembly task. To enhance the autonomy of mobile manipulation, Chen et al. [29] developed a stereoscopic vision system based on the modified iteration of the closest point algorithm to optimize the point cloud and enhance the teaching ability of the robot by using the stereoscopic vision-guided tele-operation control algorithm. Through these two functions, the mobile manipulator can learn semi-autonomously and work autonomously. In 2019, Chen et al. [30] improved the stereo vision algorithm and the iterative closest point (ICP) algorithm for object pose estimation. Based on this algorithm, the robot can select and adjust the robot’s pose, and the grasping pose relies on the object pose to maximize the maneuverability of the end-effector. With the development of deep learning, it is constantly applied to mobile operations. In reference [31], Wang et al. proposed a novel mobile manipulation system, which combines the deep reinforcement learning algorithm with visual perception. Although using the visual perception advantage of deep reinforcement learning, the robot can operate the grasping task in an unstructured environment. The effectiveness of the mobile manipulation system is verified in simulations and in the real world.

### 2.2. SLAM for Manipulation

The navigation and location of mobile robots in uncertain environments are always the basic subjects of robot research. A lot of research and reviews have been undertaken to explore the simultaneous localization and mapping (SLAM) in mobile manipulation [6,32,33,34]. Kohlbrecher et al. [35] described a Hector-SLAM-based [36] open-source SLAM system for urban search-and-rescue missions, which enables robots to map and locate themselves in a degraded urban environment, and independently explore disaster sites to identify victims and other interesting objects. Similarly, Wang et al. [37] used optical flow calculation and wavelet transformation to solve the problem of SLAM in emergency search-and-rescue environments. The three-dimensional (3D) reconstruction, based on RGB-D information, is carried out by combining a two-dimensional (2D) map and three-dimensional objects in order to realize the positioning of the robot in unfamiliar environments. In [38], Xu et al. proposed a mobile robot extensible positioning system. The system integrates a stereoscopic camera, an inertial measurement unit (IMU) and an ultra-ultra-wideband network made up of five anchors. Based on the mobile robot constantly adding new anchor points to the network and estimating anchor point location by recursive least square (RLS), the network is extended. With extended networks, the robot can determine its location in unfamiliar environments. For dynamic environments, the work of [39] presents a SLAM system that automatically projects objects into dynamic environments for semantic processing and creates a global 3D environment map to eliminate the influence of dynamic objects and unknown objects.

### 2.3. Visual Tracking Grasp

Dynamic tracking grasp is the most challenging research area in mobile manipulation. Despite the excellent results of the fixed-based grasp technique, the mobile-based grasp still faces great challenges, and researchers have conducted numerous studies on the subject. The key to dynamic tracking grasp is target tracking, especially visual-based target tracking. Zhang et al. [40] detected a target using the support vector machine (SVM) classifier, which was trained by the scale-invariant feature transform (SIFT) feature points, and used the Kalman filter to estimate the location and direction of the target. This method captures objects on the conveyor belt at a speed of 0.4 m/s. Similarly, Kim [41] developed an Opti-Track system to track the target ball, load by objects, and quickly predict the trajectory of the target and capture it, where the trajectory of the target was learned and trained by machine-learning algorithms. Furthermore, in [42], the authors proposed a method of dynamic switching between local and global planners to track and grasp moving objects. The local planner maintained the end-effector with a steady grasping posture to smoothly follow the object motion. If the previous graspable part becomes unreachable, the global planner quickly moves the end-effector to a new grasp pose to track and grasp the target. In contrast to the previous methods for tracking fast-moving objects, Ardon et al. [43] designed a fully functional grasping system based on the visual servo framework for humanoid service robots to track and grasp targets. This system uses a marker-less tracker model to track the target, and deal with the inaccurate kinematics of the robot based on a pattern tracking method for the end-effector. All of these are based on monocular camera and improve the grasp success rate by 48.8%. In [44], the authors also designed a precision assembly robot system for small components. This system adopts monocular vision guidance for dynamic grasping and two eye-to-hand cameras are used to posture alignment, where the end-effector position is compensated for according to differential motion principle.

## 3. Method

To accomplish autonomous mobile operation in a complex environment, the robot needs to reach the operating area quickly and accurately, then search and lock the target, and complete the operating task. Therefore, the mobile manipulation system needs to be composed of different subsystems, such as mobile chassis, manipulation arm, navigation and positioning systems, and environment perception and object-detection systems. To elaborate on the method we propose in this paper, the mobile manipulation system is divided into the SLAM subsystem, including the mobile chassis and navigation system, and the grasping subsystem, including the manipulation arm and perception system.

### 3.1. Enhanced Localization Based on Laser-Reflector Landmarks

Location plays an important role in mobile manipulation. In order to further improve the positioning accuracy of the mobile platform, we improved the particle filter positioning algorithm in AMCL based on the triangle matching. This idea was inspired by the method proposed by de Miguel et al. in [45]. Several modifications are made to integrate reflector information and the result of trilateration into the loop. In this section, all those modifications and adjustments are described in detail.

#### 3.1.1. Map Building and Matching Template Library Establishment

**Figure 1:**
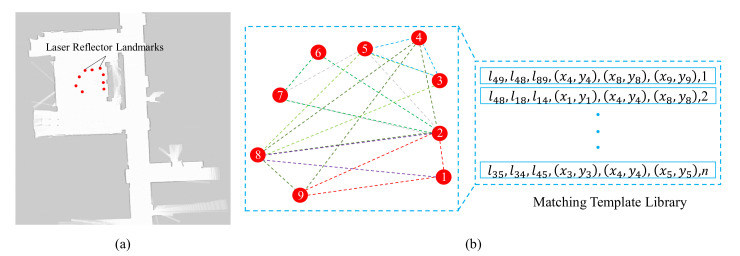
Map building and template library establishment. (**a**) Map generated by Gmapping [46], where the red dots are laser-reflector landmarks. (**b**) Establishing the matching template library M of the laser-reflector landmarks. In the template library, all items contain the side length and the vertex coordinates of the triangles and are sorted by the maximum side length.

The autonomous movement of the robot depends on reusable environmental maps. For our method, we use Gmapping [46] to build the occupancy grid map of the scenarios and mark the position of the laser reflector as a landmark point as shown in Figure 1a. We establish the global triangle-matching template library M, which contains all the triangles composed of any three landmarks in the map. Each item of M contains the side length and the vertex coordinates of the triangle. All items are sorted by the maximum side length of corresponding triangles, as shown in Figure 1b.

#### 3.1.2. Laser-Reflector Identification Based on Triangle Matching

**Figure 2:**
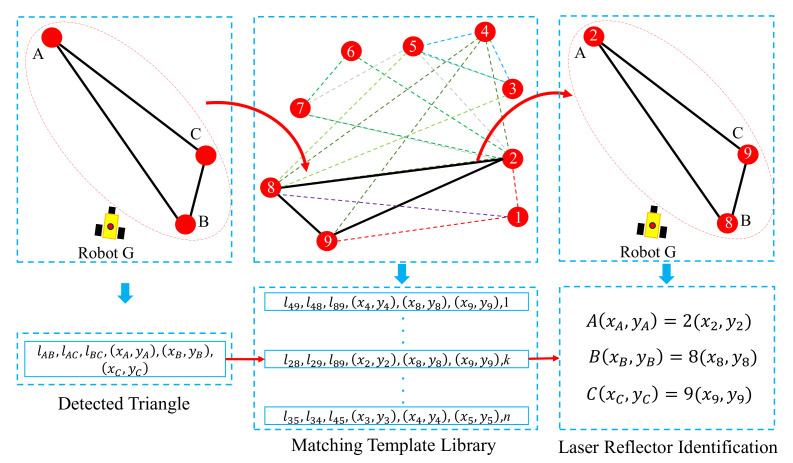
The process of triangle matching. A, B, C are the detected laser reflectors and $\left(x_{i},y_{i}\right)$ is the coordinate of the *i*-th detected laser reflector. $l_{ij}$ is the distance between the reflector *i* and *j*. G is the mobile robot. The number is the ID of the laser-reflector landmarks in matching template library M.

The mobile robot uses the detected laser reflectors to position itself, and its positioning accuracy depends on the position accuracy of the detected laser reflectors. When the robot detects the laser-reflector landmarks, it will identify their ID and obtain corresponding coordinates through triangle matching. If the number of detected laser reflectors is no less than 3, the laser-reflector identification process based on triangle matching will be activated. First, based on the received laser data (distance and orientation information), the mobile robot calculates the relative locations of the detected laser reflectors, and thus obtains the side length of the detected triangles. Then, the mobile robot searches the matched triangle in the matching template library by the side length of the detected triangle. Once all side lengths of the detected triangle match successfully with a matched triangle in the library, its coordinate is identified and added to the set of the identified laser reflectors R. Taking Figure 2 as a simple example, A, B and C are the detected laser reflectors and their coordinates are $\left(x_{A},y_{A}\right)$, $\left(x_{B},y_{B}\right)$ and $\left(x_{C},y_{C}\right)$, respectively. The detected triangle △ABC formed by these three detected laser reflectors has side lengths of $l_{AB}$, $l_{AC}$, and $l_{BC}$. Assuming $l_{AB}$ > $l_{AC}$ > $l_{BC}$, the maximum length $l_{AB}$ is first searched in M, and $l_{AC}$, $l_{BC}$ are used to match the triangle sequentially. As a result, the detected triangle △ABC matches the triangle △289 in the library. In this case, A, B and C would be identified as 2, 8 and 9, respectively.

#### 3.1.3. Position Weight Calculation

AMCL is a robot probability localization method that tracks the robot position by particle filter. In this paper, we change the weight and position of the particles to solve the problem of kidnapping and improve the location accuracy. Unlike [45], we use the estimated position of the mobile robot as input to enhance the weight of the particles in the AMCL algorithm. During navigation, the robot detects many laser reflectors and quickly determines the location of the laser reflector based on the triangle matching. The position of the robot can be estimated by the principle of trilateration.

**Figure 3:**
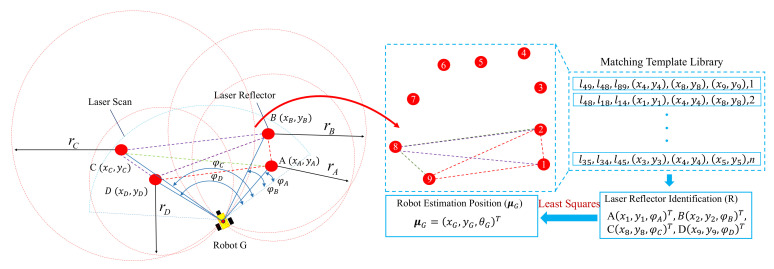
The process of positioning. A, B, C and D are the detected laser reflectors. φ and *r* are the observation angle and detection distance of the detected laser reflector, respectively. *G* is the mobile robot. $\theta_{G}$ is the orientation of the robot based on the observation angle of the detected laser reflectors. $\mu_{G}$ is the estimation position of the robot.

Assuming the robot detects *n* laser reflectors, we can obtain the ID and corresponding coordinates of each detected laser reflector by triangle matching. Therefore, the relationship between the robot and the detected laser reflectors can be described as follows:

(1) $$\left\{\begin{array}{c} {\left(x_{G}-x_{1}\right)}^{2}+{\left(y_{G}-y_{1}\right)}^{2}=r_{1}^{2}\\ {\left(x_{G}-x_{2}\right)}^{2}+{\left(y_{G}-y_{2}\right)}^{2}=r_{2}^{2}\\ \vdots \\ {\left(x_{G}-x_{i}\right)}^{2}+{\left(y_{G}-y_{i}\right)}^{2}=r_{i}^{2}\\ \vdots \\ {\left(x_{G}-x_{n}\right)}^{2}+{\left(y_{G}-y_{n}\right)}^{2}=r_{n}^{2} \end{array}\right.$$

where $\left(x_{G},y_{G}\right)$ represents the estimation position of the robot which can be obtained by solving Equation (1) with the least squares method. $\left(x_{i},y_{i}\right)$ is the position of *i*-th detected laser reflector. Moreover, the orientation of the robot can be obtained as follows:

(2) $$\theta_{G}=\left\{\begin{array}{c} \begin{array}{c} \begin{array}{cccccc} \frac{1}{n}\sum_{i=1}^{n}\left[\arctan\left(\frac{y_{i}-y_{G}}{x_{i}-x_{G}}\right)-\varphi_{i}\right] & \; & & \; & & x_{i}>x_{G} \end{array}\\ \begin{array}{cc} \frac{1}{n}\sum_{i=1}^{n}\left[\arctan\left(\frac{y_{i}-y_{G}}{x_{i}-x_{G}}\right)-\varphi_{i}+\pi \right] & y_{i}\geq y_{G},x_{i}<x_{G} \end{array} \end{array}\\ \begin{array}{cc} \frac{1}{n}\sum_{i=1}^{n}\left[\arctan\left(\frac{y_{i}-y_{G}}{x_{i}-x_{G}}\right)-\varphi_{i}-\pi \right] & y_{i}<y_{G},x_{i}<x_{G} \end{array}\\ \begin{array}{cccccccc} \frac{1}{n}\sum_{i=1}^{n}\left[\frac{\pi }{2}-\varphi_{i}\right] & \; & & \; & & \; & & y_{i}>y_{G},x_{i}=x_{G} \end{array}\\ \begin{array}{ccccccc} \frac{1}{n}\sum_{i=1}^{n}\left[-\frac{\pi }{2}-\varphi_{i}\right] & \; & & \; & & \; & y_{i}<y_{G},x_{i}=x_{G} \end{array} \end{array}\right.$$

where $\theta_{G}$ represents the orientation of the robot, φ is the observation angle of the detected laser reflectors. Therefore, the estimated pose of the robot can be expressed as $\mu_{G}=\left(x_{G},y_{G},\theta_{G}\right)$. Figure 3 shows an example of the positioning process where the mobile robot *G* detects four reflectors A, B, C and D. The estimated position of the robot can be obtained by Equations (1) and (2). And the position weight of the particle can be calculated as follows:

(3) $$d^{\left[i\right]}=\frac{1}{{\left(2\pi \right)}^{\frac{3}{2}}}\exp\left(-\frac{1}{2}{\left(x_{t}^{\left[i\right]}-\mu_{G}\right)}^{T}\left(x_{t}^{\left[i\right]}-\mu_{G}\right)\right)$$

where the position and orientation of each particle is defined as $x_{t}^{\left[i\right]}=\left(x_{i},y_{i},\varphi_{i}\right)$.

#### 3.1.4. Orientation Weight Calculation

In navigation, the accuracy of maps determines the accuracy of robot navigation. The map error often leads to location failures and the kidnapping problem. To improve the accuracy of map matching in navigation, the laser-reflector information will be used to enhance the weight of particles in the AMCL algorithm. Therefore, we compute the weight score to evaluate the accuracy of the particle matched with the map based on the Gaussian model [47]. The weight score $s_{i}$ of the particle is calculated as follows:

(4) $$s^{\left[i\right]}=\frac{1}{n}\sum_{i=1}^{n}\frac{1}{\sigma \sqrt{2\pi }}e^{\frac{-{\left(\varphi_{i}-\mu \right)}^{2}}{2\sigma^{2}}}$$

where *n* is the number of the detected laser reflectors. μ and σ represents the mean value and the standard deviation of the observation angle, respectively. With this expression, we can evaluate the particle position matched with the map and use it to modify the probability of the newly generated particles.

#### 3.1.5. Weight Adjusting and Particle Generation

In the initialization phase, a set of particles $\chi_{t}=\left\{(x_{t}^{\left[i\right]},\omega_{t}^{\left[i\right]}),i=0,1,\cdots ,N\right\}$ is randomly added. From the AMCL algorithm, the current state of the robot is predicted by the motion model $p\left(x_{t}|x_{t-1},u_{t}\right)$, where $x_{t-1}$ represents the last state of the robot and $u_{t}$ describes the control information. The predictive probability density function at time *t* can be expressed as $p\left(z_{t},\bar{M}|x_{t}^{\left[i\right]},M\right)$, where $z_{t}$ and $\bar{M}$ represent the observation information and the result of trilateration at time *t*, respectively. According to [47], the weight $\omega_{t}^{\left[i\right]}$ of each particle can be calculated as follows:

(5) $$\omega_{t}^{\left[i\right]}=\omega_{t-1}^{\left[i\right]}p\left(z_{t},\bar{M}|x_{t}^{\left[i\right]},M\right)$$

In the positioning process, the new weight of each particle can be generated as follows:

(6) $$\omega_{t-new}^{\left[i\right]}=\alpha \cdot s^{\left[i\right]}\cdot \omega_{t}^{\left[i\right]}+\beta \cdot d^{\left[i\right]}$$

The coefficients α and β are both constants and used to balance the position and the orientation. The coefficients α and β are empirically set to 154 and 1, respectively. The weights are then normalized to make the sum of all the weights equal 1.

In contrast to the AMCL algorithm which determines whether new particles are added according to the position of robot and the weight of particles, and randomly distributes the particles in the map, this paper defines a different function to generate new particles $X_{t-new}$ according to the estimated position $\mu_{G}$ and the probability of generating those new particles. The function of the newly generated particles are to follow a normal distribution centered in $\mu_{G}$.

(7) $$X_{t-new}=\lambda Q+\mu_{G}$$

where Q is a random vector with distribution N[0, 1]. The coefficients λ is empirically set to 1. In addition, the probability of generating a new particle is:

(8) $$p\left(x_{t-new}|x_{t},u_{t-1},z_{t},\bar{M}\right)=\left\{\begin{array}{c} \begin{array}{cc} d_{i}, & \mathrm{if}\;d_{i}>0.01 \end{array}\\ \begin{array}{cc} 0, & \mathrm{otherwise} \end{array} \end{array}\right.$$

In the experiment, we use the AMCL algorithm to initialize the robot. Then, the robot updates the weight of the particles based on the result of the identified laser reflectors and the estimated position of the robot. Finally, we randomly add new particles near the estimated position $\mu_{G}$ according to the weight of $d^{\left[i\right]}$, which indicates the distance between the particle position and the estimated position of the robot. This improved algorithm is shown in Algorithm 1.

**Algorithm 1** The improved adaptive Monte Carlo localization (AMCL) algorithm.
**Input:** control information $u_{t}$, observation information $z_{t}$, matched template libraries M;
**Output:** new particles set $\chi_{t}$;

1: Initialization: $R=\bar{M}=\chi_{t}=\emptyset$, $\omega_{t-new}=0$, new particle $x_{i-new}=\emptyset$, cache particle set $\bar{\chi_{t}}=\emptyset$;

2:
R,$\bar{M}$ = LaserReflectorIdentification$\left(z_{t},u_{t},M\right)$;

3:
**if**
 $\bar{M}=\emptyset$
 
**then**

4: **for**
*i* = 0 to
 
*N*
**do**

5:   
Sample
 
$x_{t}^{\left[i\right]}$∼$p\left(x_{t}|x_{t-1}^{\left[i\right]},u_{t}\right)$;//Original AMCL

6:    $\omega_{t}^{\left[i\right]}$∼$measurement\_model(z_{t},x_{t}^{\left[i\right]})$;//Original AMCL

7:   
$\bar{\chi_{t}}=\bar{\chi_{t}}+\langle x_{t}^{\left[i\right]},\omega_{t}^{\left[i\right]}\rangle$;

8:    draw
 
*i*
 
withprobability
 
∝
 
$\omega_{t-1}^{\left[i\right]}$;

9:   
add
 
$x_{t}^{\left[i\right]}$to$\chi_{t}$;

10: 
**end for**

11:
**else**

12: 
$x_{t}$ = motion_model($x_{t-1},u_{t}$);

13: **for**
  *i* = 0 to
 
*N*
**do**

14:   $d^{\left[i\right]}=PositionWeightCalculation\left(\bar{M},x_{t}^{\left[i\right]}\right)$;

15:   
$s^{\left[i\right]}=OrientationWeightCalculation\left(z_{i}\right)$;

16:   $\omega_{t-new}^{\left[i\right]}=\alpha \cdot s^{\left[i\right]}\cdot \omega_{t}^{\left[i\right]}+\beta \cdot d^{\left[i\right]}$;

17:   $\bar{\chi_{t}}=\bar{\chi_{t}}+\langle x_{t}^{\left[i\right]},\omega_{t-new}^{\left[i\right]}\rangle$;

18:  
**end for**

19:  
$\omega_{t-new}=normolaizeweight\left(\omega_{t-new}\right)$;

20:  
**for**
 
*i* = 0 to
 
*N*
**do**

21:   $p\left(x_{t-new}|x_{t},u_{t-1},z_{t},\bar{M}\right)=probofnewparticle\left(d^{\left[i\right]}\right)$;//function (8)

22:  
**if**
 
$rand\left(\right)\leq p\left(x_{t-new}|x_{t},u_{t-1},z_{t},\bar{M}\right)$
 
**then**

23:    $x_{i-new}=ParticleGeneration\left(\omega_{t-new}\right)$;

24:      add
 
$x_{i-new}$
 
to
 
$\chi_{t}$;

25:  
**end if**

26:  
**end for**

27:
**end if**

28:**return**$\chi_{t}$


### 3.2. Multiple-Object Detection and Dynamic Tracking Grasp

The ultimate goal of mobile manipulation is to operate the target object. Although methods for manipulating stationary objects or movement-known objects have been well studied [40,48], there are still great challenges for manipulating randomly placed and moving objects. Therefore, we use the object-detection model to detect and locate the object, and use the visual servo technology to control the manipulator to perform a tracking grab on the moving object. In the following section, the details regarding the object-detection model and dynamic tracking grasp are described.

#### 3.2.1. Multiple-Object Detection and Location

To manipulate multiple objects, the robot needs to identify the object and locate its position relative to the mobile manipulator. The main challenge for object detection is the possibility of multiple simultaneous object instances. The instances might vary in size, shape, attitude, and appearance. The target detection module should overcome the influence of environment on vision and detect and locate all objects in the image. In this study, we use the single-shot multi-box detector (SSD) approach, proposed by Liu in [49]. The mobile manipulation mission is a real-time task, and we choose the SSD approach since it is renowned for its accuracy and speed performance.

**Figure 4:**
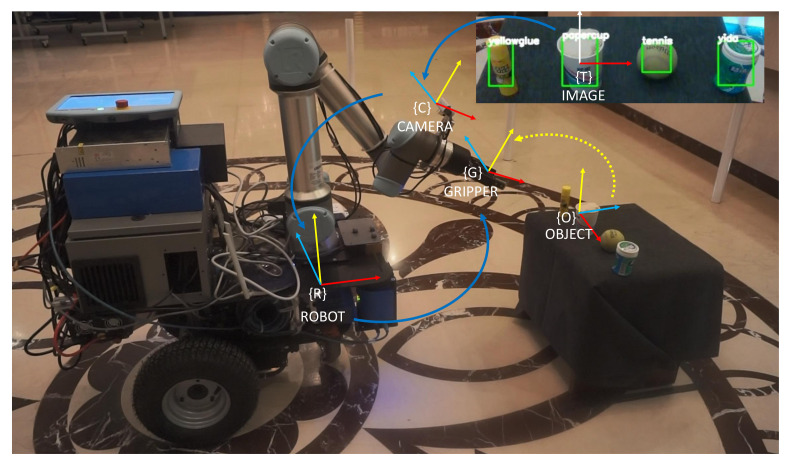
Coordinate transforming from the camera to the robot and gripper.

The object-detection module takes RGB images as input and outputs a list of detected targets. Each detection target contains a category label and the pixel coordinates of the bounding box in the image. Since the bounding box envelops the contour of the target, the center of the bounding box is regarded as the center of the object, whereas the longest side of the bounding box is regarded as the graspable edge. To obtain the 3D position of the object relative to the mobile manipulator, the pose of the object relative to the camera can be obtained by converting the pixel coordinates of the object center to the camera coordinate system and the distance is the camera depth value. Therefore, the position of the object relative to the robot can be obtained by coordinate transformation. The coordinate system transformation is shown in Figure 4.

#### 3.2.2. Dynamic Tracking and Grasping

**Figure 5:**
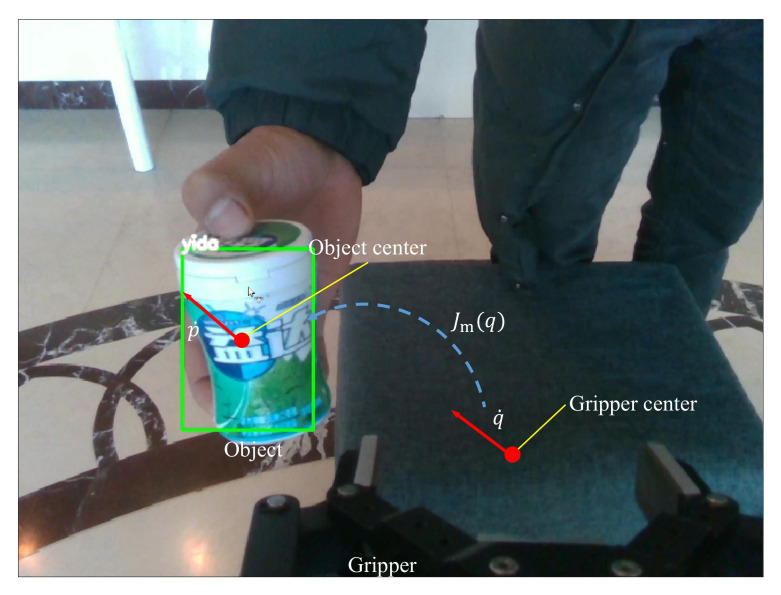
The tracking module based on visual servo.

Grasping is one of the important tasks performed in mobile manipulation, especially the grasping of randomly placed and moving objects. During the process of handling the moving object, the end-effector needs to locate and track the object in real time. In our method, we combine object detection and image-based visual servoing (IBVS) method to track and grasp the objects. From the detection module, taking the image and depth as inputs, we obtained the 2D coordinate in the image and 3D position in the robot coordinate system of the object’s center. This means that we can map the end-effector velocities to the velocities of the object center point through the Jacobian matrix, as follows:

(9) $$\dot{p}\left(t\right)=J_{m}\left(t\right)\dot{q}\left(t\right)$$

where $\dot{p}\left(t\right)$ represents the velocity of the object center and $\dot{q}\left(t\right)$ represents the end-effector velocity at *t* time, respectively. $J_{m}\left(t\right)$ is the Jacobian matrix image. Now, we can obtain the desired end-effector velocities, which are converted into the object center velocities via the inverse Jacobi or the pseudo-inverse, as shown in Figure 5.

To track a moving object in real time, we calibrate the gripper center between the fingers of the end-effector as the tracking point. Then, we use the error between the tracking point and the object center as the control parameter. Based on the error, the IBVS method is used as a closed-loop to control the tracking process. The error e(t) between the tracking point and the object center is:

(10) $$e\left(t\right)=P_{c}\left(t\right)-P_{T}\left(t\right)$$

where $P_{c}\left(t\right)$ represents the object center point; $P_{T}\left(t\right)$ represents the tracking point. We take the derivative of Function (10) and put the result into Function (9). The velocities of the end-effector can be described as:

(11) $$\dot{q}\left(t\right)=-\lambda J_{m}^{+}\left(t\right)\dot{e}\left(t\right)$$

where λ is the parameter of control gain, $J_{m}^{+}\left(t\right)$ is the pseudo-inverse and represented as follows:

(12) $$J_{m}^{+}\left(t\right)={\left(J_{m}^{T}\left(t\right)J_{m}\left(t\right)\right)}^{-1}J_{m}^{T}\left(t\right)$$

Generally, the multi-DOF manipulator has singularity, joint limit and maximum extension limit. To overcome these limitations and achieve better control of the system, we adopt two approaches. First, due to the manipulator being fixed onto the mobile platform, appropriate platform mobility can ensure that the object will be within the manipulator’s working space and can avoid the manipulator reaching the limits of its operating position. Second, we reconfigure the mobile manipulator’s configuration space in the null space to minimize these undesirable effects. Due to the multiple degrees of freedom, the arms often have a high degree of redundancy and large null spaces in which the system can reconfigure, without changing the pose of the end-effector. To address this, the Function (11) can be further rewritten as:

(13) $$\dot{q}\left(t\right)=-\lambda J_{m}^{+}\left(t\right)\dot{e}\left(t\right)+N{\dot{q}}_{0}$$

where *N* is a null space projection of the Jacobian and ${\dot{q}}_{0}$ is a set of additional end-effector velocities. Manipulability is a metric that represents the distance to a manipulator singularity in joint space. In this context, the manipulability is computed as:

(14) $$\Omega =\sqrt{\det(J_{m}\left(t\right)\left(J_{m}^{T}\left(t\right)\right)}$$

Please note that when a joint reaches a singular configuration, the Jacobian loses rank and Ω becomes zero due to a zero eigenvalue of $J_{m}\left(t\right)\left(J_{m}^{T}\left(t\right)\right)$. We maximize this metric to avoid singularities and improve control stability. In the domain of mobile manipulation, the mobile base can be used to improve manipulability than a manipulator alone. For example, the mobile base can improve the manipulator’s maneuverability by moving closer to the target, when the arm extends to the edge of its workspace.

## 4. Experiment

Mobile manipulation systems usually integrate many actuators and sensors to accomplish complex mobile operation tasks. This section focuses on the evaluation of the proposed mobile manipulation system through experimentation. First, the settings of the mobile manipulator system and the experiment environment are introduced, then the mobile operation task is defined and evaluated.

### 4.1. System Overview

#### 4.1.1. System Hardware

**Figure 6:**
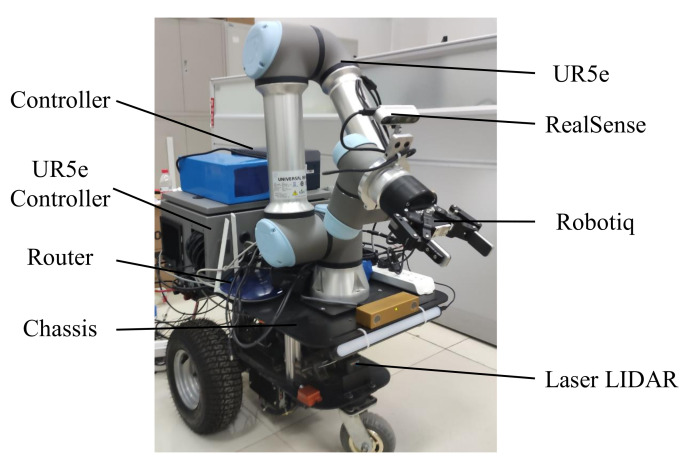
The prototype of the proposed mobile manipulator.

To evaluate the proposed mobile manipulation system, we developed a mobile manipulator, as shown in Figure 6. The mobile manipulator is composed of a mobile platform, a six-degrees-of-freedom (DOF) arm with a two-finger gripper, and multiple sensors for object detection and environmental perception. The mobile platform consists of a passive universal wheel and two active drive wheels driven by DC motor. The 6-DOF arm is UR5e, supported by Universe Robotics and mounted on the mobile platform, with a workspace of 850 mm and a maximum payload of 5 kg. The end-effector for completing the grasping task is a two-finger gripper, manufactured by Robotiq, with a stroke of 85 mm. The grip force is adjusted to be under 110 N, and the maximal payload is 5kg. The perception sensors include the LIDAR for SLAM and the RGB-D camera used for grasp. The control system of the mobile manipulator consists of an upper control system and a bottom control system. The upper control system runs on a computer equipped with an NVIDIA GPU and is responsible for the navigation control of the mobile platform and the detection and tracking grasp of the objects. The bottom control system runs on an STM32 and is responsible for calculating and executing the motion of each motor according to the upper control movement instruction. The detailed hardware parameters of the mobile manipulator are shown in Table 1.

#### 4.1.2. Control Framework

**Figure 7:**
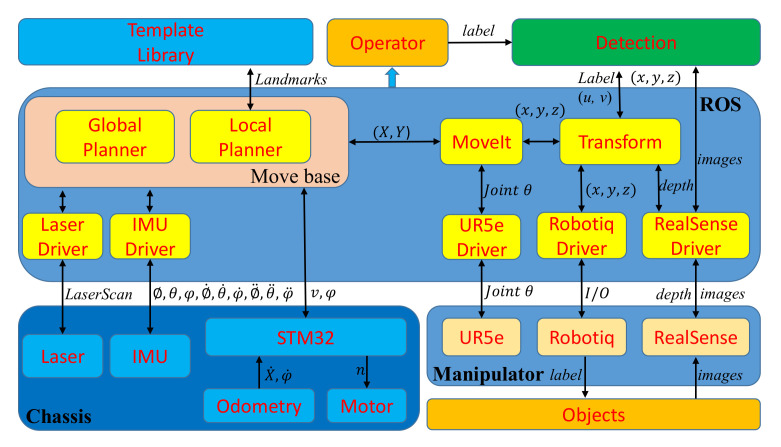
ROS-Based Control Framework.

We use the Robot Operating System (ROS) [50], which integrates the subsystems, such as the mobile chassis, sensors, and manipulator subsystems. The subsystems communicate with each other through the topic. The ROS-Based Control Framework is shown in Figure 7.

### 4.2. Experiment Setting

#### 4.2.1. Environment Description

**Figure 8:**
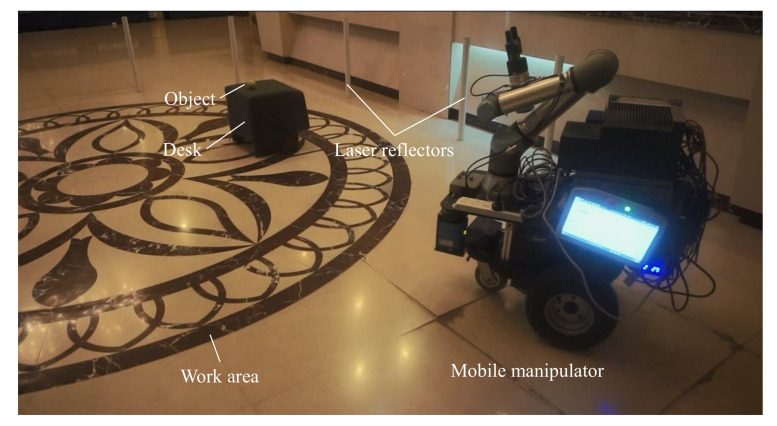
The experimental scenario.

Figure 8 shows the real environment used to evaluate the performance of the proposed mobile manipulation system in practice. The environment includes a lobby and a corridor, as shown in Figure 1. The work area is in the lobby and is surrounded by nine laser reflectors. The target objects are randomly placed on a desk. The height of the desk is 350 mm. The other environment setting is similar to the indoor environment.

#### 4.2.2. Task Definition

To evaluate the capability of the integrated mobile manipulation system, we choose a classical mobile manipulation task, i.e., a mobile picking task. This manipulation task is challenging because it requires a feasible policy that seamlessly considers both the navigation and manipulation, based on the onboard sensors. The mobile manipulator needs to be able to randomly initialize and start in the corridor, autonomously navigate to the work area, rapidly search the desk, and then accurately detect and grasp the object. The task pipeline is as follows:

(1) The mobile manipulator navigates to the work area without collisions;
(2) The mobile manipulator locates and approaches the desk;
(3) The manipulator detects and grasps the object;
(4) The manipulator dynamically tracks and grasps the moving object.

### 4.3. Experiment Results

#### 4.3.1. Localization Experiments

**Figure 9:**
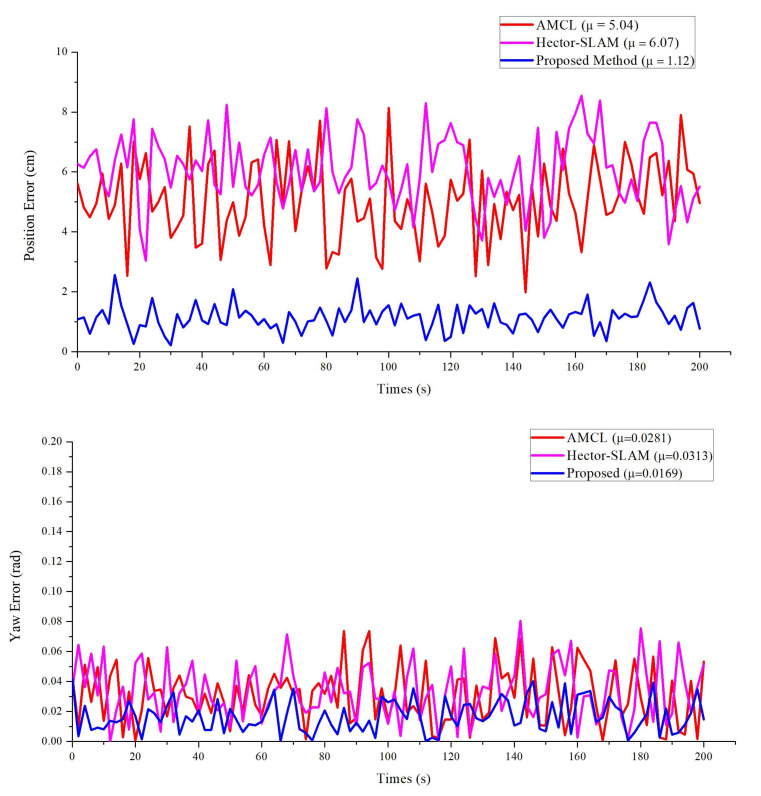
Comparison between the proposed method with AMCL and Hector SLAM. (**a**) Position Error vs. Time, (**b**) Yaw Error vs. Time.

In real experiment environments, the location of the robot is randomly initialized. The robot navigates to a target point and measures the error of the position. To evaluate the proposed method, the AMCL [5] and Hector-SLAM [35] algorithms are compared for the position tasks. As shown in Figure 9, the position error provided by our approach is better than the others and has almost the same errors for its orientation. However, compared to the other algorithms, our method has a smaller steady-state error. In 20 repeated experiments, all methods successfully navigated the mobile manipulator to the target point, but the position errors vary between methods. As shown in Table 2, the position error of our proposed approach is lower than the other methods. The position error of the proposed method is within 12 mm and the pose error is within one degree (the angle with *x*-axis). We consider a position error of less than 12 mm to be the standard for successful navigation, and our proposed method achieves a 95% success rate, which is superior to the other methods. The reason for this is that in the process of mobile manipulator movement, the weight and position of particles are changed based on the triangulation matching, the convergence of particles is accelerated, and thus the position accuracy of the mobile manipulator is improved.

**Figure 10:**
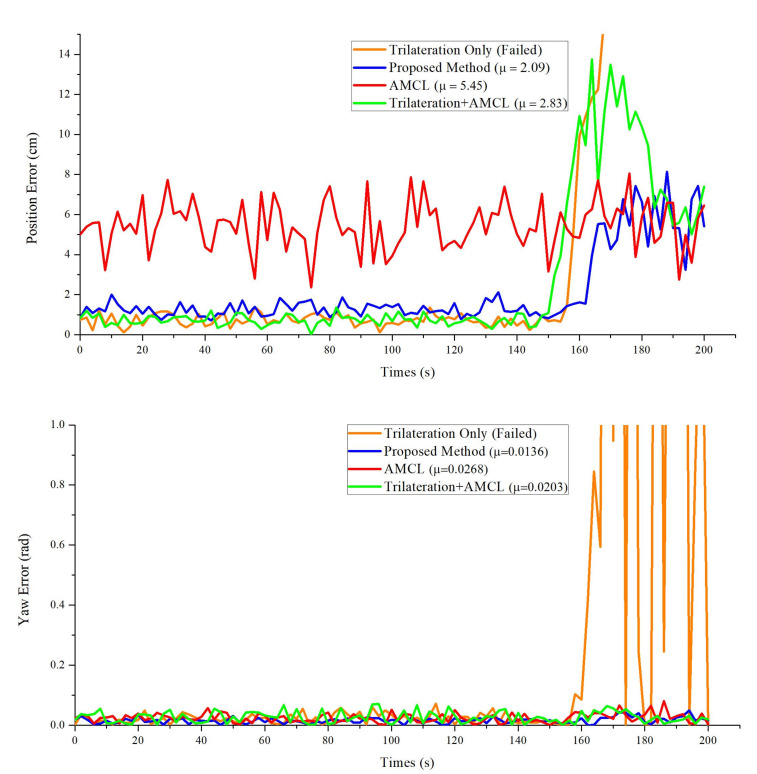
Comparison results of our proposed method with trilateration-only method, AMCL and T-AMCL. (**a**) Position Error vs. Time, (**b**) Yaw Error vs. Time.

In real scenarios, the situation that less than three reflectors can be detected frequently occurs due to the movement of people or obstacles. To further evaluate the proposed method, AMCL, the trilateration-only method and the method combining trilateration and AMCL (T-AMCL), are compared. As shown in Figure 10, when the robot detects less than three laser reflectors (around 150 s), the trilateration-only method failed and the T-AMCL method took about 25 s to re-initialize and converge, thus reducing the positioning accuracy and robustness. However, our proposed method is to improve the positioning accuracy by improving the particle weights in AMCL. In this case, the particles can be directly applied to the AMCL algorithm and help the robot get out of trouble quickly. Therefore, our proposed method is more robust in dynamic complex environments.

#### 4.3.2. Multiple-Object Grasp Experiments

**Figure 11:**
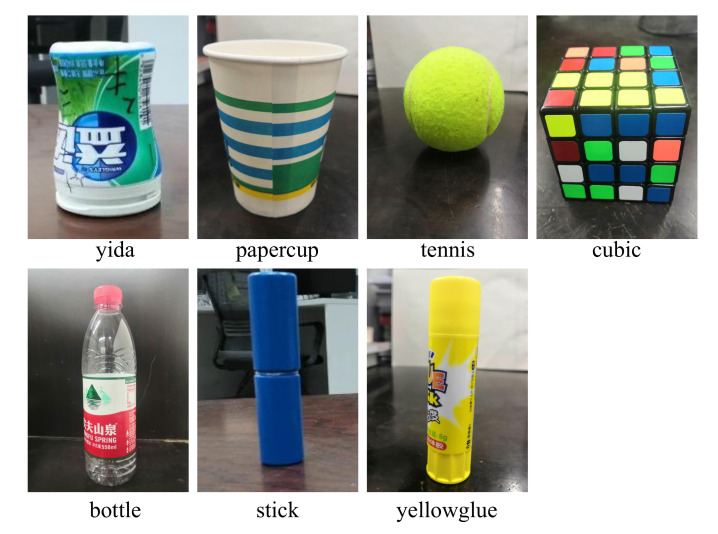
The seven different objects.

**Figure 12:**
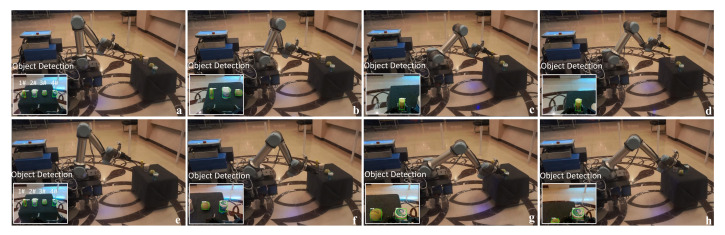
The process of multi-object grasping. (**a**) Detecting the multiple objects(1# yellow glue, 2# paper cup, 3# tennis, 4# yida). (**b**) Approaching the 1# object. (**c**,**d**) Grasping the 1# object. (**e**) Detecting the 4# object. (**f**) Approaching the 4# object. (**g**,**h**) Grasping the 4# object.

**Figure 13:**
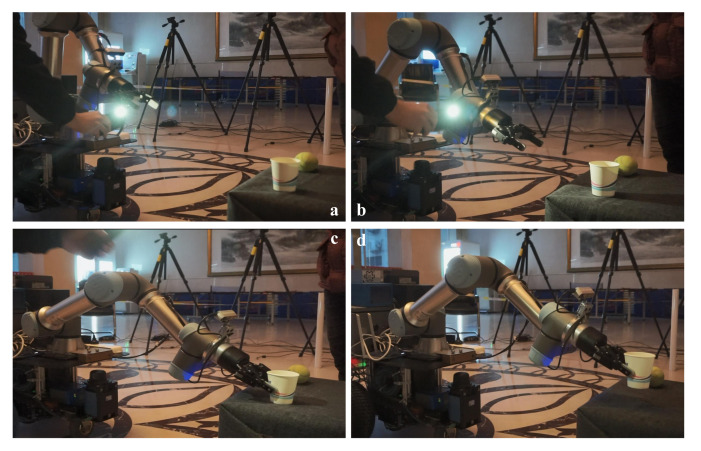
The process of grasping in a high-light environment. (**a**) Detecting the object. (**b**) Approaching the object. (**c**) Grasping the object. (**d**) Placing the object.

The speed and accuracy of object detection determines the success of mobile manipulation tasks. However, the most time-consuming and memory-consuming part is object detection. To address this issue, we simplify the object-detection model by using the state-of-the-art algorithm NetAdapt [51] to reduce memory consumption. For the trained model, NetAdapt automatically and iteratively identifies and removes redundant channels from the model to reduce the computational complexity. In addition, histogram homogenization, edge detection and mean filtering are used to preprocess the image before being sent into the detection module. This method improves the success rate of target detection in dark environments. In the experiment, we choose three light conditions and seven different objects, such as yida bottle, tennis, paper cup and so on, as shown in Figure 11. For the different objects, the yida bottle and water bottle have a cylinder shape, the yellow glue and the stick are have a slim cylinder shape with different textures, the tennis ball has a ball shape, and the cube has a cube shape. These objects are good representations of most common objects. Table 3 shows the grasp success rates in different light situations with various objects. The result shows that the system achieves a higher success rate, except for the bottle, which is bigger than the others and could lead to unstable grasping. Because blue is a difficult color to be detected in a dark environment, the success rate of detecting the stick is the lowest. The performance of non-reflective objects is better in the high-light environment and comparative in the dark environment compared with the reflective objects. Figure 12 shows the successful detection and grasping for the multiple objects and Figure 13 shows the process of detecting and grasping in the high-light environment. All of these operations can be done in real-time.

#### 4.3.3. Dynamic Tracking Grasp Experiments

**Figure 14:**
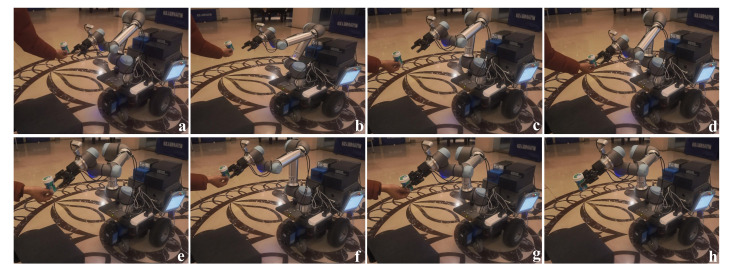
The process of dynamic tracking grasp. (**a**) Detecting the object. (**b**,**c**) Approaching the object. (**d**–**f**) Dynamically tracking the object. (**g**,**h**) Grasping the object.

In some situations, the mobile platform needs to deal with a moving object. As such, the manipulator needs to have the ability to dynamically track and grasp the object. As described in Section 3.2, the dynamic tracking grasp systems are combined with the object detection and visual servo. In the tracking grasp experiment, the object is moved randomly by the operator and the manipulator tracking the object depends on the object-detection module. The dynamic tracking grasp process is shown in Figure 14. The result shows that the manipulator can track and grasp the object well when the object velocity is normal (*v* < 0.1 m/s). However, when the object moves fast, the manipulator fails to track the object and stops moving, waiting for the target to reappear in order to continue tracking.

#### 4.3.4. Integrated System Experiments

**Figure 15:**
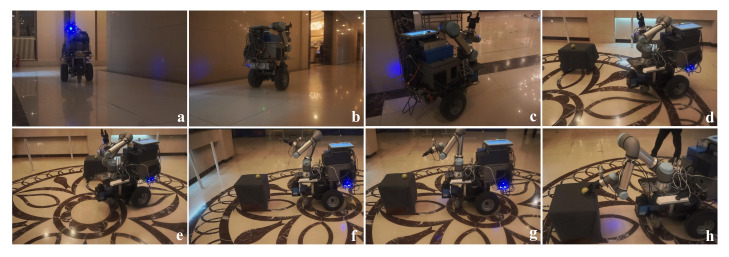
The process of autonomous mobile manipulation. (**a**) Robot initiating. (**b**,**c**) Navigating to the target area. (**d**,**e**) High-precision localization based on laser reflectors. (**f**) Detecting the desk. (**g**) Detecting the object. (**h**) Grasping.

To evaluate the integrated system, the mobile manipulator is assigned to execute a complex mobile manipulation task of object grasp. First, the position of the robot is randomly initialized in the corridor. Then, the operator sends the object name. Next, the mobile manipulator navigates to the target area and completes the high-precision self-location and search of the target desk by rotating. Then, the manipulator detects and returns a list of results to the operator and autonomously grabs the target object. Some of the robot navigation and grasping process are shown in Figure 15. Table 4 summarizes the success rates of mobile manipulation with three kinds of objects. Compared with other objects, the papercup has the lowest grasping success rate due to its soft structure, while the tennis ball has the highest detection and grasp success rate, because its spherical shape is easy to be recognized and grasped.

#### 4.3.5. Discussion

Our experiments demonstrate the effectiveness of the proposed method on a mobile robot. The mobile manipulator can automatically navigate to the target area and locate itself with high-precision, and then detect and grasp the object based on vision. For the moving object, based on visual servo, it can dynamically track and grasp the object in real time. However, some problems exist. On the one hand, the positioning accuracy depends on the accuracy of the position of the laser reflectors. Although the robot can achieve high-precision positioning based on laser-reflector information, if the position of laser-reflector changes, the matched template library needs to be reconstructed. On the other hand, if we combine object detection with the visual servo, the closed-loop control and dynamic tracking grasp of the manipulator are realized. To grasp the objects precisely, the performance of the proposed method should be improved, which is a subject of our future work.

## 5. Conclusions

In this paper, a mobile manipulation system is proposed, which integrates navigation, high-precision positioning, object detection and dynamic tracking grasp. Based on the triangle matching with the laser-reflector data, the mobile robot can achieve high-precision positioning in a target area. The object-detection model can detect multiple objects under different lighting conditions. The closed-loop control of manipulator and the dynamic tracking control of moving targets are realized by combining target detection with visual servo. Finally, experiments in the real world demonstrate the effectiveness of the proposed method. The mobile manipulator can autonomously complete mobile manipulation tasks in a real environment, and can achieve a high success rate of 90% for localization and 95% for grasping.

In future research, we will pay more attention to the intelligence and stability of robots in mobile operation. First, we will study the practical application of visual navigation in mobile robots, further integrating visual information with laser data, or replacing laser data to improve the robot’s ability to perceive environmental data. Second, we would like to study the object pose estimation method based on vision and apply it to the mobile manipulation. Finally, we will further study the cooperative motion of the mobile chassis and the mechanical arm, and solve the problem of occlusion in the grasping process, and apply it to more complex mobile manipulation tasks and complex environments.

## Figures and Tables

**Table 1:** The hardware parameters of the mobile manipulator.

Parameter	Value
Computer	I7-9700, 16G, 512SSD
GPU	GTX 1070, NVIDIA
LIDAR	NAV350-3232, SICK
RGB-D Camera	RealSense d435i, Intel
Manipulator	UR5e, UR
Gripper	2F-85 Gripper, Robotiq
Battery	48V 32AH
Chassis size (L × W × H)	810 mm × 493 mm × 671 mm

**Table 2:** The result of the localization

Methods	Position Mean Error (cm)			Pose Mean Error (rad)	Success Rate
	*x*	*y*	Position	Theta	Position Error < 1.1 cm
AMCL [5]	3.91	3.32	5.14	0.027	0.20
Hector-SLAM [35]	4.63	3.84	6.12	0.032	0.35
Ours	0.82	0.73	1.09	0.017	0.90

**Table 3:** Mobile manipulation success rate for different objects in different light conditions

Object Type	Light Conditions		
	High-Light	Normal	Dark
yida	0.90	0.95	0.80
yellowglue	0.80	0.90	0.80
tennis	0.95	0.95	0.70
cubic	0.85	0.90	0.75
bottle	0.75	0.85	0.75
stick	0.85	0.90	0.50
papercup	0.90	0.90	0.65

**Table 4:** Mobile manipulation success rate for different objects (total = 20)

Object Type	Success Rate		
	Navigation	Detection	Grasping
yida	0.85	0.88	0.93
tennis	0.95	0.95	0.94
papercup	0.90	0.89	0.88
